# Predictive Models of Mortality for Hospitalized Patients With COVID-19: Retrospective Cohort Study

**DOI:** 10.2196/21788

**Published:** 2020-10-15

**Authors:** Taiyao Wang, Aris Paschalidis, Quanying Liu, Yingxia Liu, Ye Yuan, Ioannis Ch Paschalidis

**Affiliations:** 1 Department of Electrical and Computer Engineering Boston University Boston, MA United States; 2 Department of Biomedical Engineering Boston University Boston, MA United States; 3 Center for Information and Systems Engineering Boston University Boston, MA United States; 4 Brown University Providence, RI United States; 5 Department of Biomedical Engineering University of Science and Technology Shenzen China; 6 Third People’s Hospital of Shenzhen Second Hospital Affiliated to Southern University of Science and Technology Shenzen China; 7 School of Artificial Intelligence and Automation Huazhong University of Science and Technology Wuhan China

**Keywords:** coronavirus, COVID-19, mortality, Wuhan, China, machine learning, logistic regression, support vector machine, predictive modeling

## Abstract

**Background:**

The novel coronavirus SARS-CoV-2 and its associated disease, COVID-19, have caused worldwide disruption, leading countries to take drastic measures to address the progression of the disease. As SARS-CoV-2 continues to spread, hospitals are struggling to allocate resources to patients who are most at risk. In this context, it has become important to develop models that can accurately predict the severity of infection of hospitalized patients to help guide triage, planning, and resource allocation.

**Objective:**

The aim of this study was to develop accurate models to predict the mortality of hospitalized patients with COVID-19 using basic demographics and easily obtainable laboratory data.

**Methods:**

We performed a retrospective study of 375 hospitalized patients with COVID-19 in Wuhan, China. The patients were randomly split into derivation and validation cohorts. Regularized logistic regression and support vector machine classifiers were trained on the derivation cohort, and accuracy metrics (F1 scores) were computed on the validation cohort. Two types of models were developed: the first type used laboratory findings from the entire length of the patient’s hospital stay, and the second type used laboratory findings that were obtained no later than 12 hours after admission. The models were further validated on a multicenter external cohort of 542 patients.

**Results:**

Of the 375 patients with COVID-19, 174 (46.4%) died of the infection. The study cohort was composed of 224/375 men (59.7%) and 151/375 women (40.3%), with a mean age of 58.83 years (SD 16.46). The models developed using data from throughout the patients’ length of stay demonstrated accuracies as high as 97%, whereas the models with admission laboratory variables possessed accuracies of up to 93%. The latter models predicted patient outcomes an average of 11.5 days in advance. Key variables such as lactate dehydrogenase, high-sensitivity C-reactive protein, and percentage of lymphocytes in the blood were indicated by the models. In line with previous studies, age was also found to be an important variable in predicting mortality. In particular, the mean age of patients who survived COVID-19 infection (50.23 years, SD 15.02) was significantly lower than the mean age of patients who died of the infection (68.75 years, SD 11.83; *P*<.001).

**Conclusions:**

Machine learning models can be successfully employed to accurately predict outcomes of patients with COVID-19. Our models achieved high accuracies and could predict outcomes more than one week in advance; this promising result suggests that these models can be highly useful for resource allocation in hospitals.

## Introduction

The ongoing pandemic due to the novel coronavirus SARS-CoV-2 has caused worldwide disruption; national governments have imposed drastic measures to contain the pandemic, and the global economy has been impacted [1]. SARS-CoV-2 causes a disease called COVID-19, which is marked by symptoms such as cough, fever, chills, and a range of respiratory symptoms [2]. As of the end of July 2020, the total number of confirmed cases of COVID-19 had surpassed 15 million, and the total number of deaths was approaching 650,000 [3,4].

As the virus continues to proliferate, governments, institutions, and hospitals have struggled to allocate resources such as tests, hospital beds, intensive care unit beds, and ventilators. A significant amount of work has already been performed to predict and track the spread of the virus [3-8]. Recent and ongoing efforts are being made to understand the biomarkers and comorbidities associated with severe COVID-19 disease [9-12]. This work has been important in helping hospitals to classify patients in terms of risk. However, infrastructure to predict hospitalization, mortality, or other patient outcomes is lacking. Predicting these outcomes is essential, as it enables clinicians to make informed decisions regarding patients at risk. For example, clinicians can ensure that the proper resources are allocated to patients who are more likely to require critical care and the use of ventilators.

Using blood samples from patients from Tongji Hospital in Wuhan, China, we used supervised machine learning methods to predict mortality following hospitalization. These machine learning models have been used frequently in the literature for a variety of applications. Some examples include predicting the death of patients with sepsis [13,14], identifying patients at high risk of emergency hospital admissions [15], predicting hospitalization due to heart disease [16,17], and predicting diabetes complications [18,19].

The aim of this retrospective cohort study was to develop accurate models to predict mortality among hospitalized patients with COVID-19 using basic demographics and easily obtainable laboratory data.

## Methods

### Data Collection

Data were collected between January 10 and February 18, 2020, from patients admitted to Tongji Hospital in Wuhan, China. Data collection was approved by the Tongji Hospital Ethics Committee. The records collected included epidemiological, demographic, clinical, and laboratory results as well as mortality following infection with COVID-19. Data originating from pregnant and breastfeeding women or patients aged younger than 18 years and records with more than 20% missing data were excluded from the analysis [20].

### Preprocessing

Prior to model development, several preprocessing measures were undertaken. Variables were standardized by subtracting the mean and dividing by the standard deviation. Variable elimination was performed to reduce the complexity of the resulting model, improve the out-of-sample performance, and enhance the interpretability. Redundant variables and variables with more than 30% missing data were removed. In addition, we computed pairwise Spearman correlations between variables and removed one of the variables if the absolute correlation coefficient was >0.8. Furthermore, missing data in the remaining variables were imputed using the median values of the respective variables. This measure enabled us to include as many patients as possible in our analysis and is a well-documented and popular method of inferring missing values.

### Model Development

Data from a total of 375 patients were used to develop the models. These patients were split into two groups to obtain a training set and validation set. The training set was used to train and develop the models, and the validation set was used to determine the accuracy of each model. Unless otherwise noted, 70% of the data were reserved for the training set, and the other 30% were reserved for the validation set. After the data were split into training and validation sets, feature selection was performed to remove several variables. Models were trained using the training set and tested on the validation set. This process was repeated five times, and the average performance and its SD were calculated.

Feature selection was performed using ℓ_1_-norm regularization and recursive feature elimination with cross-validation. Specifically, we performed ℓ_1_-regularized logistic regression (LR) and obtained the coefficients of the model. We then eliminated the variable with the smallest absolute coefficient and performed the LR again to obtain a new model. We continued this iteration to select a model that maximizes a metric equal to the mean performance minus its SD in a validation data set.

### Model Selection

Two different types of regularized models were used in this analysis: ℓ_1_-regularized logistic regression (L1LR) models and ℓ_1_-regularized support vector machine (L1SVM) models. The models were initially fit to patient data that were collected at any time during the patients’ length of stay at the hospital. However, due to the possibility that some laboratory tests were performed close to the patients’ outcomes (death or survival), the models were also fit to patient data obtained ≤12 hours after admission. By doing this, we could ensure that the patients’ outcomes were predicted as far in advance as possible.

LR, in addition to prediction, provides the likelihood associated with the predicted outcome, which can be used as a confidence measure in decision making.

### Model Performance

The performance of the models was evaluated by calculating the weighted F1 score on the validation set. The weighted F1 score is defined as the weighted mean of the F1 score of the positive and negative classes, where the F1 score is defined as the harmonic mean of the precision and the recall. The precision, or positive predictive value (PPV), can be expressed as the ratio of the true positives to the sum of the true positives and false positives. The recall is the true positive rate (ie, the ratio of the true positives to the sum of the true positives and false negatives). The weighted F1 score, unlike the F1 score, considers all the possible outcomes (in this case, survival or death). This can combat potential class imbalance issues and evaluate whether the model accurately predicts mortality and survival, both of which are important in our context. In particular, while identifying patients with higher mortality risk can help direct more resources and attention to those patients, identifying patients who are not at risk is also helpful and can free up resources and time that would otherwise be spent on these lower-risk patients. In addition to the weighted F1 score, we also determined the PPV and the negative predictive value (NPV); the latter is defined as the ratio of the true negatives to the predicted negatives, or the precision of the negative class.

Furthermore, to gain additional insight into the roles of specific variables, we developed a “binarized” counterpart to our sparse LR model. Specifically, we defined a threshold for each variable (using the normal range of the variable) and devised a model in which each variable was either 0 (normal) or 1 (abnormal). For this model, we computed the odds ratio (OR) for each variable; this quantifies how the odds of mortality are scaled by the variable being normal vs abnormal while controlling for the remaining variables.

### Statistical Power and External Validation

To assess whether our study cohort size was sufficiently large for the models we derived, we conducted a multiple logistic regression power analysis [21]. This analysis tests the null hypothesis that a specific variable has an LR coefficient equal to zero vs the coefficient value obtained by the model. We set the Type I error probability to 0.05 and the Type II error probability to 0.2 (statistical power of 0.8), from which we obtained a minimum sample size for the variable.

Further, to demonstrate that our models are generalizable, we validated our models on a multicenter external data set. This data set contained data from 432 patients from Shenzhen, China, and 110 patients from Wuhan, China. The data set contained very limited information, encompassing the results of three laboratory tests, the times of the laboratory tests, the discharge time, and the outcome for each patient. Given this limited information, we were only able to validate our best-performing L1SVM model, which uses these three laboratory test values.

## Results

### Patient Demographics and Laboratory Tests

Table S1 in Multimedia Appendix 1 details patient demographics in addition to various laboratory values for the full patient population. The average age of the patients was 58.83 years (SD 16.46). The mean age of the patients who survived COVID-19 infection (50.23 years, SD 15.02) was significantly lower than the mean age of the patients who did not survive the infection (68.75 years, SD 11.83; *P*<.001). The proportion of men (224/375, 60%) and women (151/375, 40%) in the study cohort was similar. However, more male patients succumbed to infection (126/174, 72%, *P*<.001).

Several laboratory tests were found to have statistically different values among patients who survived and died of COVID-19 infection. Patients who succumbed to infection had LDH values that were roughly 4 times larger than those of patients who survived (755.58 compared to 215.77, *P*<.001). Patients who died also had significantly smaller percentages of lymphocytes and eosinophils in their blood (*P*<.001). Furthermore, the mean level of hs-CRP in patients who died was significantly higher than that in patients who survived (*P*<.001).

As detailed in the Methods section, two different approaches were used to model the data. The first approach was to use blood test results obtained throughout the patients’ length of stay at the hospital. Although this approach ensured that there were few missing data points, some of the blood samples were tested close to the patients’ outcomes (death or discharge from the hospital). To predict a patient’s outcome in advance, a second approach was developed using laboratory test results that were obtained ≤12 hours after the patients’ admission to hospital.

### Models Using All Laboratory Tests

We first present the results of our predictive models using all laboratory tests. These models were developed as noted in the Methods section. Of the 375 total patients, 24 (6.4%) had incomplete measurements and were omitted, leaving a total of 351 patients (93.6%) for model development. The accuracies of the models using all patient laboratory tests were determined on the validation and external test sets described in the Methods. Complete lists of all the models and their accuracies are provided in Table S2 and Table S3 in Multimedia Appendix 1.

The best-performing models were the ℓ_1_-regularized logistic regression model using 4 variables selected by recursive feature selection (L1LR 4) and the ℓ_1_-regularized support vector machine model using 3 variables selected by recursive feature selection (L1SVM 3). The L1LR 4 model had a weighted F1 score of 96.98% (SD 0.93%) on the validation set, while the L1SVM 3 model had a score of 97.36% (SD 1.10%). The L1SVM 3 model had a weighted F1 score of 94.55% on the external test set of Shenzhen and Wuhan patients.

The L1LR 4 model had an average validation PPV of 97.61% and an average validation NPV of 96.31%. The L1SVM 3 model had a similarly high average PPV and NPV of 98.27% and 96.71%, respectively. On the multicenter external test set, the accuracy of the L1SVM 3 model remained high (94.55%). Furthermore, both models used a small number of variables in their predictions.

The variables used in each of the best-performing models and the corresponding weight of each variable are reported in Table 1. The logistic regression model used four variables: lactose dehydrogenase (LDH), an enzyme that is found in most living cells and is typically released when there is tissue damage; the percentage of lymphocytes, a class of immune molecules that are found in the body; hypersensitive C-reactive protein (hs-CRP), a protein that is often used as an indication of heart disease and shows increased levels with inflammation and infection; and albumin, which is one of the main proteins found in blood and is important in regulating the pressure of red blood cells as well as transporting nutrients, proteins, and other molecules. The L1SVM 3 model used the same variables, with the exception of albumin.

**Table 1:** Coefficients showing the weights of the variables for the two best models.

Variable	Coefficient	
	L1LR 4^a^	L1SVM 3^b^
LDH^c^	1.35	1.44
Percentage of lymphocytes	–0.86	–0.47
hs-CRP^d^	0.74	0.34
Albumin	-0.64	N/A^e^

^a^L1LR 4: ℓ_1_-regularized logistic regression model using 4 variables selected by recursive feature selection.

^b^L1SVM 3: ℓ_1_-regularized support vector machine model using 3 variables selected by recursive feature selection.

^c^LDH: lactose dehydrogenase.

^d^hs-CRP: hypersensitive C-reactive protein.

^e^N/A: not applicable.

The coefficients obtained by both methods are comparable because the variables were standardized. Therefore, a larger absolute coefficient indicates that the corresponding variable is a more significant predictor. A positive coefficient implies a positive correlation with the outcome, while a negative coefficient implies a negative correlation. Of the variables selected by our models, LDH was considered to be the most important (binarized L1LR 4 OR 55.62, 95% CI 11.41-270.97). The next most important variables were the percentage of lymphocytes (binarized L1LR 4 OR 32.17, 95% CI 5.99-172.90) and hs-CRP (binarized L1LR 4 OR 13.12, 95% CI 3.65-47.23). Finally, the L1LR model found that albumin was important in predicting mortality (binarized L1LR 4 OR 4.08, 95% CI 1.45-11.48). To calculate these ORs, we used a binarized model with the following thresholds: LDH values ≥250 were set to 1, and values <250 were set to 0; lymphocyte percentage values <20 were set to 1, and values ≥20 were set to 0; hs-CRP values ≥10 were set to 1, and values <10 were set to 0; albumin values <34 were set to 1, and values ≥34 were set to 0.

As outlined in the Methods section, a power analysis was performed for the L1LR 4-variable model, and the results indicated that our sample size of 351 patients was sufficient. Specifically, this power analysis indicated that the sufficient numbers of patients to find the LR coefficient were 21 for LDH, 63 for hs-CRP, 61 for the percentage of lymphocytes, and 162 for albumin.

In addition to the previously mentioned models, we also trained models with several important variables removed. More specifically, we removed LDH, albumin, and D-D dimer, a protein that is produced by the degradation of a blood clot. The accuracies of these models were slightly lower than those of the models that included these factors. Furthermore, as we removed more variables, the accuracy of the models decreased. The validation accuracy of the L1LR model with LDH removed was 94.90% (SD 2.13%), the validation accuracy of the L1LR model with LDH and albumin removed was 94.51% (SD 2.19%), and the validation accuracy of the L1LR model with LDH, albumin, and D-D dimer removed was 94.14% (SD 2.5%) (Multimedia Appendix 1 Table S2). The models highlighted several other important factors that were not previously indicated to be important, such as the activity of prothrombin, a protein used in blood clot formation; the platelet count – the count of one of the main cells that makes up blood clots; and age. After these variables were removed, the two most important factors were hs-CRP and the percentage of lymphocytes. When fitting a model to the data using only these two factors, the validation accuracy of the model was 94.87% (SD 1.76%).

### Models Using Test Results Obtained ≤12 Hours After Admission

To predict the outcome of a patient with COVID-19 soon after admission to the hospital, we developed several L1SVM models using laboratory test results obtained no later than 12 hours after admission. More specifically, we first performed an ℓ_1_-regularized logistic regression to perform feature selection and then fed the selected features into an ℓ_1_-regularized support vector machine model. The average time between admission and the time the laboratory test was conducted was 8.4 hours (SD 2.6 hours). Furthermore, the average time between the time of the laboratory test and the patient outcome was 11.5 days (SD 7.5 days).

Table 2 details the average F1 scores and SDs for a select number of the models developed based on data collected ≤12 hours from admission. Table S4 in Multimedia Appendix 1 reports the variables selected by these models. For all models, the L1SVM was performed five times and optimized using a validation set. Of the 375 total patients, 114 (30.4%) had missing data and were excluded, leaving 261 patients (69.6%) for analysis. For these 261 patients, 90% of the data were used for training and 10% of the data were kept as a validation set. As before, the models were fit using all the variables, a limited number of variables, and all variables other than LDH, albumin, and D-D dimer.

All the models performed well, with accuracies >89% and SDs <5%. The number of variables used in each model varied greatly. The L1SVM All model used 18 of the variables provided in the data set, the L1SVM 7 model used 7 variables, the L1SVM model without LDH and albumin used 10 variables, and the L1SVM model without LDH, albumin, and D-D dimer used 12 variables. Of these models, the model that used 7 variables (including LDH, albumin, and D-D dimer) performed the best, with an accuracy of 94.08% (SD 1.81%). When LDH and albumin were removed from the model, the accuracy decreased by approximately 4%.

These L1SVM models highlighted several key variables that were not indicated by the models that included all laboratory tests. In the models that used all variables, LDH and hs-CRP were consistently two of the most important markers. However, the percentage of lymphocytes found in the blood did not appear to be consistently important in this set of models. Interestingly, the number of neutrophils, a different class of immune marker, in the blood was deemed to be an important variable.

**Table 2:** Performance of select models developed based on data collected ≤12 hours after admission.

Model	Validation set weighted F1 score (%), mean (SD)
L1SVM all^a^	90.39 (3.25)
L1SVM 7^b^	94.08 (1.81)
L1SVM no LDH^c^, albumin^d^	89.65 (4.30)
L1SVM no LDH, albumin, D-D dimer^e^	89.64 (4.89)

^a^L1SVM all: ℓ_1_-regularized support vector machine model developed using all the variables in the data set.

^b^L1SVM 7: ℓ_1_-regularized support vector machine model using 7 variables.

^c^LDH: lactate dehydrogenase.

^d^L1SVM no LDH, albumin: ℓ_1_-regularized support vector machine model developed using all variables except LDH and albumin.

^e^L1SVM no LDH, albumin, D-D dimer: ℓ_1_-regularized support vector machine model developed using all variables except LDH, albumin, and D-D dimer.

## Discussion

### Principal Findings

Our developed L1LR and L1SVM models were able to accurately predict the outcomes of patients with COVID-19, as validated by their weighted F1 scores as high as 97%. In general, the models that used laboratory test results from the duration of the patients’ hospital stays were more accurate than models that were restricted to laboratory test results obtained ≤12 hours after admission. However, even when the data were restricted, our models achieved accuracies as high as 94%. These models are more useful because they make predictions upon admission of the patient and thus provide sufficient lead time for making decisions regarding staffing and resource allocation. Because the length of stay of most patients was >1 week, our models can predict a patient’s outcome more than one week in advance, with accuracies exceeding 90%.

In many ways, our patient cohort represented a typical cohort of hospitalized patients with COVID-19. In particular, individuals who die of the infection tend to be older and male [22-25]. However, the rate of mortality in our study cohort was higher; close to 50% of the patients admitted to hospital died (174/375, 46.4%). This is likely due to the fact that Tongji Hospital admitted higher numbers of patients with severe and critical disease in Wuhan, China.

The performance of the L1SVM model using all patient laboratory tests on an external multicenter data set suggests that our models are generalizable. The performance of the model decreased by <3% when tested on the external data set compared to the validation set. This indicates that our model could be used by other hospitals worldwide to better understand the risk associated with each patient with COVID-19.

Of particularly importance was the ability of the models to perform well with a small number of predictors. Moreover, the models still performed well when certain key predictors, such as LDH, albumin, and D-D dimer, were removed due to these variables’ tendency to exhibit abnormalities at a very late stage of the disease when the outcome is inevitable. The ability of the models to perform well even with few variables can prove particularly useful, as this facilitates interpretation. Furthermore, this ability ensures that predictions can be made even when the outcome is not apparent to a sufficiently experienced physician.

In a recent study, a predictive model was developed based on a few key variables [20]. Different machine learning methods were used in this study, and a decision tree was created. The authors found that LDH, percentage of lymphocytes, and hs-CRP were important predictors of mortality; we also found these three variables to be important. The study’s models were very accurate, with F1 scores of approximately 95%. The key difference in our study is that we used laboratory test results obtained ≤12 hours after admission and tested the robustness of the models to the absence of several key variables. Therefore, we are confident that our models can accurately predict patient outcomes well in advance, in the absence of key variables, and even when the outcome may not be obvious to a trained physician.

### Limitations

One of the main limitations of this study was the relatively targeted study cohort used to derive the models. These patients lived in Wuhan, China, which was the original epicenter of the outbreak of the novel coronavirus SARS-CoV-2. However, one of our models was validated on an external multicenter cohort of patients from Wuhan and Shenzhen; this suggests that this model can be generalized to other patient cohorts, especially in China. It is less clear how well the models generalize to cohorts in other countries, where patient characteristics and care practices may differ.

### Conclusions

We developed multiple state-of-the-art supervised machine learning models to predict the outcome of infection with the novel coronavirus SARS-CoV-2. We were able to predict mortality with greater than 90% accuracy, and we identified several important predictors of mortality.

## Appendix

Multimedia Appendix 1 Select patient demographics and laboratory test results, performance of all logistic regression and SVM models evaluated on all laboratory test results, and variables and coefficients of select models evaluated using laboratory test results obtained within 12 hours of admission.

## Abbreviations

hs-CRP: hypersensitive C-reactive protein
L1LR: ℓ1-regularized logistic regression
L1SVM: ℓ1-regularized support vector machine
LDH: lactate dehydrogenase
LR: logistic regression
NPV: negative predictive value
OR: odds ratio
PPV: positive predictive value
