# Supplementary material for: Predictive Models of Mortality for Hospitalized Patients With COVID-19: Retrospective Cohort Study
Source: JMIR Med Inform. 2020 Oct 15;8(10):e21788. doi: 10.2196/21788 (PMC7572117; doi:10.2196/21788)
Supplement: Multimedia Appendix 1 [file medinform_v8i10e21788_app1.docx]

## Predictive Models of Mortality for Hospitalized COVID-19 Patients: Retrospective Cohort Study

Taiyao Wang,^1^ Ph.D., Aris Paschalidis,^2^ Quanying Liu, ^3^ Ph.D., Yingxia Liu,^4^ M.D., Ye Yuan, ^5^ Ph.D., and Ioannis Ch. Paschalidis,^1,*^ Ph.D.

^1^Department of Electrical and Computer Engineering, Department of Biomedical Engineering, and Center for Information and Systems Engineering, Boston University, Boston, MA

^2^Brown University, Providence, RI

^3^Department of Biomedical Engineering, Southern University of Science and Technology. Shenzhen, 518000, China

^4^The Third People’s Hospital of Shenzhen, Second Hospital Affiliated to Southern University of Science and Technology, Shenzhen, 518000, China

^5^Huazhong University of Science and Technology, Wuhan, China

^*^Corresponding Author

Ioannis Ch. Paschalidis,

Department of Electrical and Computer Engineering,

and Department of Biomedical Engineering,

Boston University

8 Saint Mary’s St.,

Boston, MA 02215

USA

e-mail: yannisp@bu.edu

http://sites.bu.edu/paschalidis

Tel: 617-353-0434

Fax: 617-353-0190

## Multimedia Appendix

This is a Multimedia Appendix to a full manuscript published in the J Med Internet Res. For full copyright and citation information see [http://dx.doi.org/10.2196/jmir. 21788](http://dx.doi.org/10.2196/jmir.%2021788)

**Table S1.** Select patient demographics and laboratory tests.

| Characteristic | | Survived (n=201) | Deceased (n=174) | All patients (N=375) | *P* value^a^ |
| --- | --- | --- | --- | --- | --- |
|  | |  |  |  |  |
| Age, mean (SD) | | 50.23 (15.02) | 68.75 (11.83) | 58.83 (16.46) | <.001 |
| **Gender, n (%)** | |  |  |  | <.001 |
|  | Male | 98 (49) | 126 (72) | 224 (60) |  |
|  | Female | 103 (51) | 48 (28) | 151 (40) |  |
| Lactate dehydrogenase (U/L), mean (SD) | | 215.77 (65.00) | 755.58 (442.87) | 466.24 (407.00) | <.001 |
| Percent lymphocyte, mean (SD) | | 25.69 (10.31) | 6.50 (6.17) | 16.78 (12.89) | <.001 |
| Hypersensitive C-reactive protein (mg/L), mean (SD) | | 12.54 (25.19) | 120.42 (80.88) | 62.60 (79.17) | <.001 |
| Albumin (g/L), mean (SD) | | 36.68 (3.90) | 28.10 (4.95) | 32.70 (6.15) | <.001 |
| D-D dimer, mean (SD) | | 1.00 (1.78) | 11.37 (8.91) | 5.81 (8.07) | <.001 |
| Neutrophil count, mean (SD) | | 4.24 (2.40) | 12.06 (7.12) | 7.87 (6.47) | <.001 |
| Percent eosinophils, mean (SD) | | 1.50 (1.40) | 0.14 (0.38) | 0.87 (1.25) | <.001 |
| Red blood cell distribution width, mean (SD) | | 12.59 (1.05) | 13.87 (2.10) | 13.18 (1.74) | <.001 |
| Serum chloride, mean (SD) | | 101.32 (3.56) | 105.47 (9.78) | 103.25 (6.20) | <.001 |
| Glucose, mean (SD) | | 6.40 (3.04) | 10.71 (5.99) | 8.40 (5.12) | <.001 |
| Calcium, mean (SD) | | 2.20 (0.10) | 1.98 (0.17) | 2.10 (0.18) | <.001 |
| Prothrombin activity, mean (SD) | | 95.49 (11.75) | 65.49 (21.79) | 81.57 (22.76) | <.001 |
| Platelet count, mean (SD) | | 249.27 (85.67) | 127.91 (90.95) | 192.96 (106.88) | <.001 |
| Length of stay, mean (SD) | | 11.43 (5.26) | 5.13 (3.96) | 8.51 (5.65) | <.001 |

^a^P values were calculated using the two-sided t-test for binary variables and a Kolmogorov-Smirnoff test for multi-category or continuous variables. The former tests the null hypothesis that the means of the variable in the two groups were the same, whereas the latter compares the empirical distributions of the two groups. A P value of less than 0.05 indicates that the null hypothesis should be rejected, and the alternative hypothesis accepted, implying that the values of the variable in the two groups are statistically different.

**Table S2.** Performance of all logistic regression models evaluated on all laboratory tests.

| Model | Validation set weighted F1-score (%), mean (SD) |
| --- | --- |
|  |  |
| L1LR all^a^ | 95.66 (1.85) |
| L1LR 4^b^ | 96.98 (0.93) |
| L1LR no LDH^c^ | 94.90 (2.13) |
| L1LR no LDH, albumin^d^ | 94.51 (2.19) |
| L1LR no LDH, albumin, D-D dimer^e^ | 94.14 (2.50) |

^a^L1LR all: ℓ_1_-regularized logistic regression model developed using all the variables in the data set.

^b^L1LR 4: ℓ_1_-regularized logistic regression model utilizing 4 variables.

^c^L1LR no LDH: ℓ_1_-regularized logistic regression model developed using all variables but LDH.

^d^L1LR no LDH, albumin: ℓ_1_-regularized logistic regression model developed using all variables but LDH and albumin.

^d^L1LR no LDH, albumin, D-D dimer: ℓ_1_-regularized logistic regression model developed using all variables but LDH, albumin, and D-D dimer.

**Table S3.** Performance of all SVM models evaluated on all laboratory tests.

| Performance on validation set | L1SVM all^a^ | L1SVM 3^b^ |
| --- | --- | --- |
|  |  |  |
| Weighted F1-score (%), mean (SD) | 94.14 (2.50) | 97.36 (1.10) |

^a^L1SVM all: ℓ_1_-regularized SVM model developed using all the variables in the data set.

^b^L1SVM 3: ℓ_1_-regularized SVM model utilizing 3 variables.

**Table S4.** Variables and coefficients of select models evaluated using laboratory tests within 12 hours of admission.

| L1SVM 7^a^ | | L1SVM no LDH, albumin, D-D dimer^b^ | |
| --- | --- | --- | --- |
| Variable | Coefficient | Variable | Coefficient |
|  |  |  |  |
| LDH | 0.54 | Number of Neutrophils | 0.54 |
| Age | -0.46 | Age | 0.51 |
| Number of Neutrophils | 0.3 | Aspartate aminotransferase | 0.5 |
| Hs-CRP | 0.28 | N-terminal pro b-type natriuretic peptide | 0.49 |
| Eosinophil Percent | -0.14 | Red Blood Cell Distribution Width | 0.42 |
| Red Blood Cell Distribution Width | 0.14 | Hs-CRP | 0.42 |
| Chlorine | -0.10 | Hemoglobin | 0.41 |

^a^L1SVM 7: ℓ_1_-regularized SVM model utilizing 7 variables.

^b^L1SVM no LDH, albumin, D-D dimer: ℓ_1_-regularized SVM model developed using all variables but LDH, albumin, and D-D dimer.
